# Neddylation inhibitor MLN4924 enhances H3K18 lactylation *via* binding to LDH and downregulates ITGB4 to block metastasis

**DOI:** 10.1016/j.jbc.2025.110575

**Published:** 2025-08-08

**Authors:** Hongfei Yu, Qiyin Zhou, Yongxia Chen, Changxin Zhong, Tingting Fu, Xiufang Xiong, Feng Zhu, Linbo Wang, Yi Sun

**Affiliations:** 1 Cancer Institute, The Second Affiliated Hospital, Zhejiang University School of Medicine, Hangzhou, China; 2 Institute of Translational Medicine, Zhejiang University School of Medicine, Hangzhou, China; 3 Research Center for Life Science and Human Health, Binjiang Institute of Zhejiang University, Hangzhou, China; 4 Department of Breast Surgery, Sir Run Run Shaw Hospital, Zhejiang University School of Medicine, Hangzhou, China; 5 College of Pharmaceutical Sciences, The Second Affiliated Hospital, Zhejiang University School of Medicine, Hangzhou, China; 6 State Key Laboratory of Advanced Drug Delivery and Release Systems, Zhejiang University, Hangzhou, China; 7 State Key Laboratory of Transvascular Implantation Devices, The Second Affiliated Hospital, Zhejiang University School of Medicine, Hangzhou, China

**Keywords:** MLN4924, H3K18 lactylation, ITGB4, breast cancer, metastasis

## Abstract

MLN4924, a small molecule neddylation inhibitor and a potent anticancer agent, was previously shown to have some neddylation-independent effects. Whether MLN4924 regulates histone lactylation in neddylation-dependent or independent manner is previously unknown. We reported here that MLN4924 significantly increased the lactate levels by activating lactate dehydrogenase (LDH) activity *via* inducing LDH tetramerization to promote histone H3K18 lactylation in breast cancer cells. Through combined analyses of cleavage under target & tagmentation, RNA-seq, and CHIP-PCR, we identified integrin ITGB4 as a downstream target, subjected to downregulation by MLN4924-induced H3K18 lactylation, occurred at the first intron of the ITGB4 gene. This MLN4924-mediated dose- and time-dependent ITGB4 downregulation is independent of its neddylation inhibition but can be largely abrogated by siRNA-based *LDH* knockdown or treatment with oxamate, a small molecular inhibitor of LDH. Biologically, MLN4924 effectively suppresses the migration and invasion of breast cancer cells *in vitro* and metastasis *in vivo*, which is largely rescued by ITGB4 overexpression. Taken together, our study revealed a new mechanism by which MLN4924 suppresses the migration and invasion of breast cancer cells by epigenetically inhibiting ITGB4 expression *via* enhancing H3K18 lactylation.

Neddylation, a posttranslational protein modification similar to ubiquitylation, involves a cascade of enzymatic reactions in which the ubiquitin-like protein NEDD8 is covalently attached to lysine residues of substrate proteins *via* the sequential reaction of the NEDD8-activating enzyme (NAE/E1), NEDD8-conjugating enzyme (E2), and NEDD8 ligase (E3) (1, 2). Unlike ubiquitylation, which is mainly involved in substrate degradation by proteasome, neddylation regulates the stability and subcellular localization of substrates, thus modulating various biological processes (3, 4). Numerous studies have shown that the neddylation pathway is hyperactivated in various types of human cancers and is associated with poor prognosis (3, 5).

MLN4924, also known as pevonedistat, is the first NAE small molecule inhibitor targeting the NAE (6). NAE is a heterodimer composed of regulatory subunit amyloid precursor protein-binding protein 1 (also known as NAE1) and catalytic subunit UBA3 (ubiquitin-activating enzyme 3, also known as NAEβ). In the presence of ATP and Mg^2+^, neddylation is initiated by NAE, which catalyzes the formation of thioester bond between NEDD8 and Cys216 at the enzymatic active site of UBA3. By forming the NEDD8-MLN4924 adduct at the active site of NAE, MLN4924 blocks subsequent enzymatic reaction, thus inhibiting NAE activity and the entire neddylation reaction (7). In many preclinical studies, MLN4924 has demonstrated potent anticancer activity in a variety of human cancer cells and are currently in multiple phase I/II clinical trials as an anticancer agent in combination with chemotherapeutic drugs (3, 5).

In addition to blocking neddylation, emerging evidence has shown that MLN4924 exerts neddylation-independent effects (8). For examples, our previous studies have shown that (1) MLN4924 promoted epidermal growth factor receptor dimerization, thereby activating the RAS/MAPK and PI3K/AKT pathways to enhance tumor cell spheroid formation and EGF-driven wound healing at the low concentrations (9); (2) MLN4924 induced PKM2 tetramerization to promote glycolysis, and MLN4924 anticancer effect was enhanced when it was combined with a glycolysis inhibitor (10); Other studies have shown that (3) MLN4924 inhibited ACT1-TRAF6 interactions, attenuated IL-17A-mediated NF-κB activation, and reduced lung inflammation (11); (4) MLN4924 inhibited IRF3 binding to the IFN-β promoter to suppress IFN-β production (12); and (5) MLN4924 activated the JNK signaling pathway to downregulate c-FLIP levels and sensitize cells to TRAIL-induced apoptosis (13). These findings underscore the complex regulatory network regulated by MLN4924 and highlight the importance of further investigating its mechanisms of action to maximize its clinical applications.

Lactate, traditionally regarded as a byproduct of cellular metabolism, has recently emerged as a critical regulator of tumor biology (14, 15). Lactate was reported to serve as an energy substrate through mitochondrial oxidation in cancer cells, to suppress immune cell cytotoxicity, to promote the M2 polarization of tumor-associated macrophages, and to facilitate angiogenesis in the tumor microenvironment (16, 17). More importantly, lactate was recently found to act as a precursor to induce lactylation modifications on histone lysines, thus playing a pivotal role in epigenetically regulating gene expression (18). Since then, an increasing number of studies have revealed that histone lactylation serves as a critical regulatory mechanism in proliferation and drug resistance of cancer cells. For examples, elevated H3K18 lactylation in melanoma cells enhanced YTHDF2 expression to promote proliferation of cancer cells (19). H3K18 lactylation in bladder cancer cells upregulated YBX1 and YY1 to confer resistance to cisplatin (20). In clear cell renal cell carcinoma, VHL inactivation promotes H3K18 lactylation, which in turn enhances the expression of platelet-derived growth factor receptor β, driving proliferation and metastasis of cancer cells (21).

In this study, we followed up our previous observation that MLN4924 significantly elevated extracellular lactate levels in breast cancer cells (10) and reported that MLN4924 enhanced lactate production by activating lactate dehydrogenase (LDH) *via* triggering its tetramerization, leading to enhanced H3K18 lactylation. We identified ITGB4, a critical mediator of cancer cell migration and invasion, as a downstream target, whose expression was suppressed *via* MLN4924-mediated H3K18 lactylation. Our study revealed a novel neddylation-independent mechanism by which MLN4924 suppressed the migration and invasion of breast cancer cells *in vitro* and metastasis *in vivo via* epigenetically targeting ITGB4.

## Results

### MLN4924 enhances lactate production by promoting LDH activity via inducing its tetramerization

**Figure 1:**
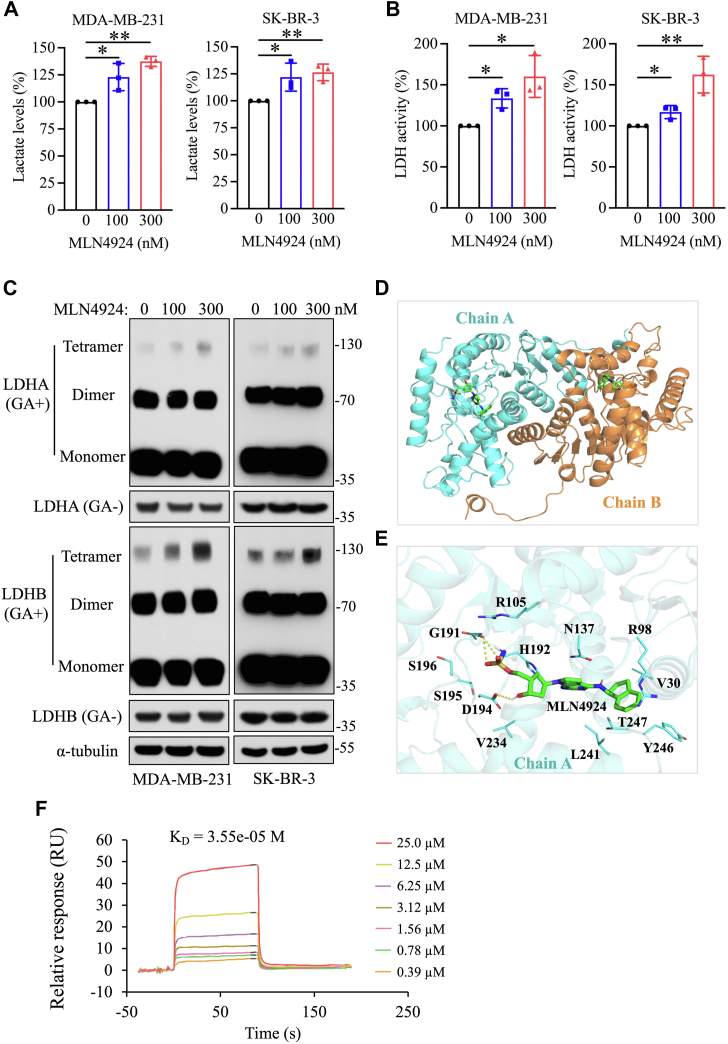
**MLN4924 enhances lactate production by promoting LDH activity and tetramer formation.***A*, changes of intracellular lactate levels after MLN4924 treatment for 24 h in breast cancer cells (mean ± SD, Mann–Whitney U test, n = 3). *B*, changes of lactate dehydrogenase (LDH) activity after MLN4924 treatment for 24 h in breast cancer cells (mean ± SD, Mann–Whitney U test, n = 3). *C*, detection of LDH tetramer levels in breast cancer cells treated with MLN4924 for 24 h. Cell lysates were prepared using nonreducing lysis buffer, crosslinked with 0.05% glutaraldehyde (GA) at 37 °C for 3 min, followed by immunoblotting. *D*, the docking pose of MLN4924 binding in the LDH dimer. *E*, the representative structure of MLN4924 binding in the pocket during the MD simulation. The MLN4924 were shown in *stick*, important resides were shown in *lines*, and hydrogen bonds were shown as *yellow dashed lines*. *F*, SPR analysis of LDHA and MLN4924. ∗*p* < 0.05, ∗∗*p* < 0.01. MD, molecular dynamics; SPR, surface plasmon resonance.

To investigate the effect of MLN4924 on lactate metabolism, we first measured the changes in lactate levels in two breast cancer cell lines, MDA-MB-231 and SK-BR-3, and found that the intracellular lactate levels were significantly increased upon MLN4924 treatment for 24 h (Fig. 1*A*). To elucidate the underlying mechanism, we measured the effect of MLN4924 on LDH, an enzyme that catalyzes the reaction from pyruvate to lactate. Indeed, MLN4924 significantly increased LDH enzymatic activity in a dose-dependent manner (Fig. 1*B*). Importantly, MLN4924 had no effect on the protein levels of two LDH subunits, LDHA and LDHB (Fig. S1*A*), indicating the regulation occurred at the level of enzymatic activity.

Given that LDH operates as a tetrameric form, composed of two subunits of LDHA and LDHB, which are assembled into either homotetramers or heterotetramers for catalytic activity (22), we then explored whether MLN4924 affects the tetramerization of LDHA or LDHB. Through the cross-linking (GA^+^)-coupled immunoblotting analysis, we observed a significant increase in the formation of LDHA and LDHB tetramers upon MLN4924 treatment (Fig. 1*C*). Thus, MLN4924 activates LDH catalytic activity is due to the induction of their tetramerization, leading to increased lactate production.

We next determined how MLN4924 triggers LDH tetramerization through molecular docking and simulation. From the crystal structure (PDB ID: 6Q0D) (23), the LDH tetramer is composed of four identical monomers (331 residues) or two dimers. We, therefore, evaluated the effect of MLN4924 on protein structural stability based on the monomer, as well as its impact on the stability of the polymerization interface based on the dimer. Interestingly, MLN4924 was docking into the binding pocket of LDH (Fig. 1*D*). For the monomer, the root mean square deviation and root mean square fluctuation values of the ligand-bound (Complex) system were less fluctuating than those of the unbound (Apo) system during the 100 ns molecular dynamics (MD) simulations (Fig. S1, *B* and *C*), indicating that MLN4924 binding can stabilize the protein structure. For the dimer, the root mean square deviation and root mean square fluctuation values of the Complex system were also less fluctuating that those of the Apo system (Fig. S1, *B* and *C*), suggesting that MLN4924 binding facilitates the stabilization of the LDH dimeric structure. In addition, the representative structure showed that MLN4924 binds to a binding pocket defined by residues R105, H192, N137, R98, T247, Y246, V30, L241, V234, D194, S195, 196, and G191 (Fig. 1*E*). The ligand forms hydrogen bonds with G191 and D194 and hydrophobic interactions with V234, L241, and V30. Thus, it appears that MLN4924 interaction with residues within the binding pocket stabilizes both the primary protein structure and the structural polymerization, contributing to the stabilization of the LDH tetrameric structure.

To validate the direct interaction between MLN4924 and LDH, we performed a surface plasmon resonance (SPR) assay using purified recombinant LDHA protein and MLN4924. The results revealed a dissociation constant 3.55e-05 M, indicating a strong binding affinity (Fig. 1*F*). In addition, we carried out a cellular thermal shift assay, which showed that MLN4924 treatment decreased the accumulation of LDHA (Fig. S1*D*). Together, these findings provide compelling evidence for a direct interaction between MLN4924 and LDHA.

### MLN4924 promotes histone lactylation independent of neddylation inhibition

**Figure 2:**
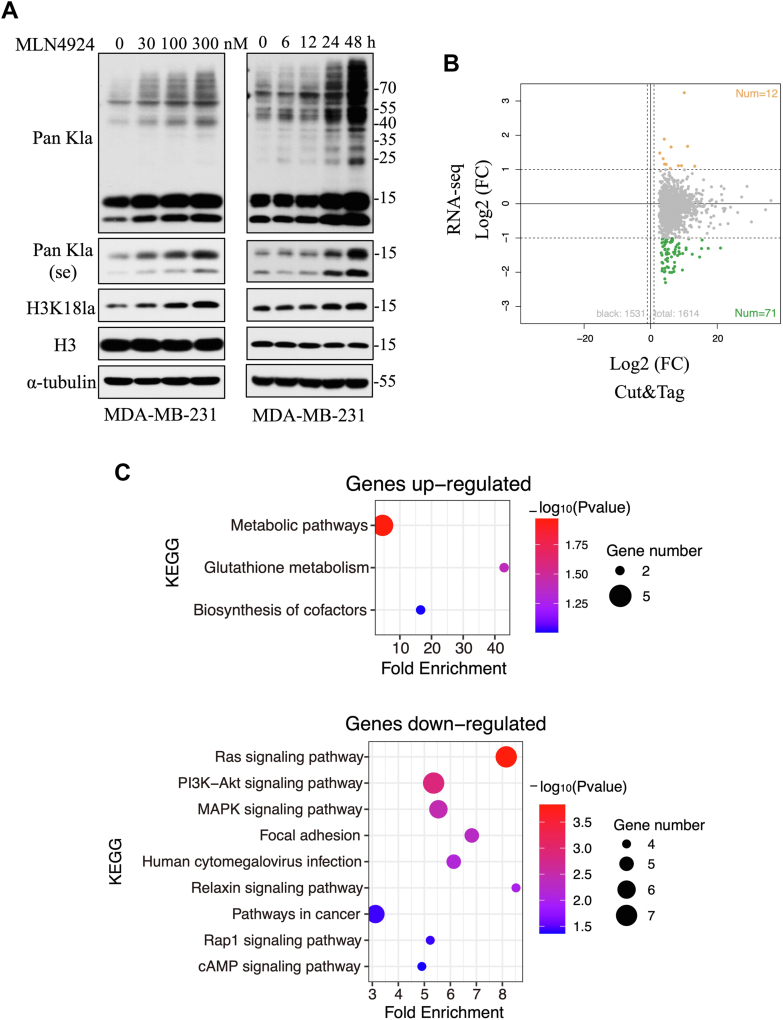
**MLN4924 promotes histone lactylation and modulates downstream gene expression.***A*, MLN4924 promotes lactylation in dose- and time-dependent manner. MDA-MB-231 cells were treated with indicated concentrations of MLN4924 for 24 h or indicated time points with 100 nM MLN4924, followed by immunoblotting. Pan-Kla detects lactylation at all lysine sites on proteins. The two lower bands represent lactylated histones, while the upper bands represent lactylated nonhistone proteins. se: short exposure, only lactylated histones were shown in the short-exposure bands. *B*, integrated analysis of transcriptome sequencing and CUT&Tag sequencing results before and after MLN4924 treatment of MDA-MB-231 cells. *C*, Kyoto Encyclopedia of Genes and Genomes pathway analysis to reveal upregulated (*top*) and downregulated genes (*bottom*). CUT&Tag, cleavage under target & tagmentation.

A recent study showed that lactate acted as a precursor for lysine lactylation on histones, thereby epigenetically regulating gene expression (18). We investigated the effects of MLN4924 on lysine lactylations in MDA-MB-231 and SK-BR-3 breast cancer cells and found that MLN4924 promoted in a dose- and time-dependent manner lysine lactylations in both nonhistone and histone (H3K18) proteins (Figs. 2*A* and S2*A*). To determine whether this lactylation effect is dependent of its neddylation inhibition, we used siRNA-based silencing approach to knockdown UBA3, the catalytic subunit of NAE, to which MLN4924 inhibits and found that UBA3 knockdown had minimal, if any, effect on the lactylations of nonhistone proteins and H3K18 (Fig. S2*B*), suggesting a neddylation-independent effect of MLN4924 on lysine lactylation. Furthermore, the siRNA-based knockdown of Cullin-1, a major neddylation substrate, also did not alter the protein lactylation levels (Fig. S2*C*).

### MLN4924 epigenetically regulates gene expression via H3K18 lactylation

We next determined biochemical consequence of MLN4924-induced H3K18 lactylation. Two sets of samples with or without MLN4924 treatment of MDA-MB-231 cells were prepared. One set was for RNAseq to identify genes with altered expression, whereas another set was subjected to cleavage under target & tagmentation (CUT&Tag) sequencing using an H3K18la-specific antibody to pinpoint the genes with elevated H3K18 lactylation. Integrative analysis of two approaches identified 83 overlapping genes with 12 genes significantly upregulated, whereas 71 genes significantly downregulated, following MLN4924 treatment (Fig. 2*B* and Table S1). Kyoto Encyclopedia of Genes and Genomes enrichment analysis revealed that the upregulated genes were involved in "metabolic pathways", “glutathione metabolism,” and “biosynthesis of cofactors”, while the downregulated genes were predominantly associated with Ras, PI3K-Akt, MAPK signaling pathways, and "focal adhesion" pathways (Fig. 2*C*).

### MLN4924 suppresses ITGB4 expression via H3K18 lactylation

To further explore the target genes affected by MLN4924-induced H3K18 lactylation, we selected seven upregulated and 10 downregulated genes with functional importance for confirmation and analysis. Quantitative PCR results confirmed that the transcription levels of most selected candidate genes changed upon MLN4924 treatment in a dose-dependent manner, consistent with the sequencing data (Fig. S3*A*). We next performed rescue experiments to assess whether inhibition of lactate production would reverse MLN4924 effect using both pharmacological (with oxamate, a LDH inhibitor) and genetic (siRNA-based knockdown of LDHA/LDHB) approaches. We found that among 17 genes tested, ITGB4, a well-known gene involving tumor migration and invasion (24), was the only gene whose downregulation by MLN4924 was significantly rescued by both approaches, while a minimal rescue or rescue only by one approach was observed in the other 16 genes (Fig. S3, *B* and *C*).

**Figure 3:**
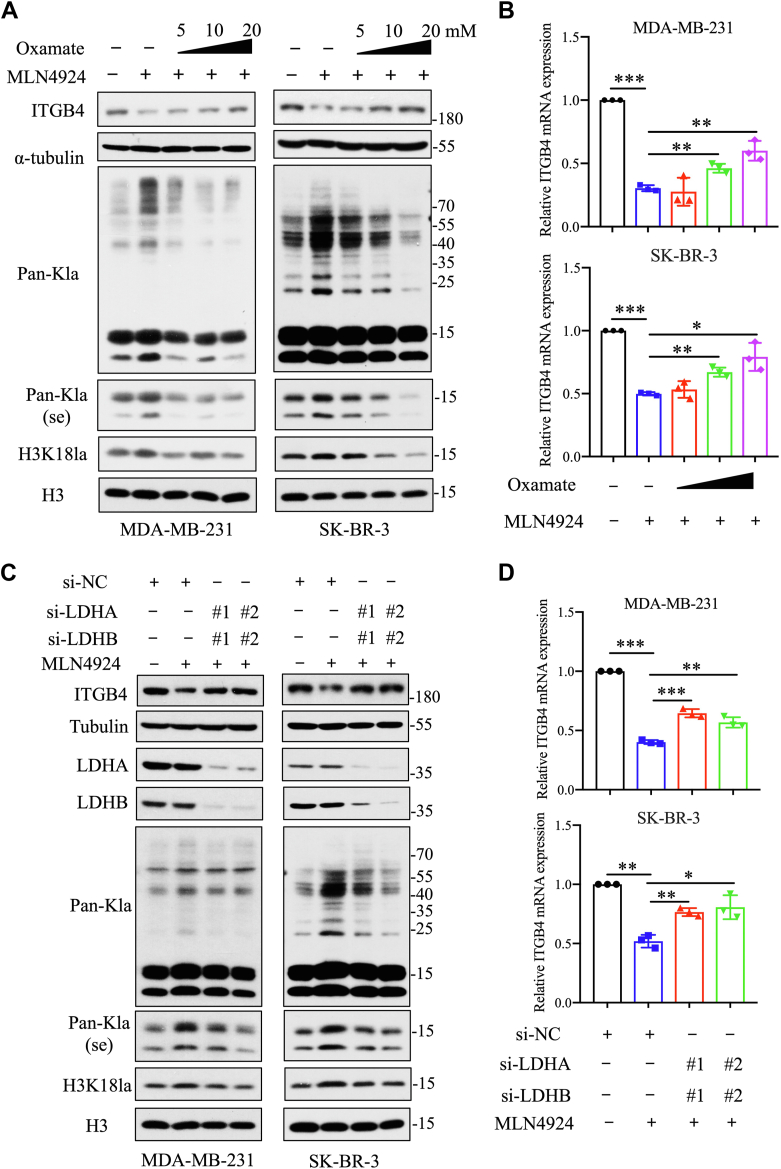
**MLN4924 inhibits ITGB4 expression by activating LDH.***A*, immunoblot analysis of lactylation levels and ITGB4 protein levels in breast cancer cells treated with MLN4924 and oxamate. *B*, qPCR analysis of ITGB4 mRNA expression in breast cancer cells treated with MLN4924 and oxamate (mean ± SD, Mann–Whitney U test, n = 3). *C*, immunoblot analysis of lactylation levels and ITGB4 protein levels in breast cancer cells after transfecting siRNA targeting LDHA and LDHB for 48 h and MLN4924 treatment for 24 h. *D*, qPCR analysis of ITGB4 mRNA expression in breast cancer cells after transfecting siRNA targeting LDHA and LDHB for 48 h and MLN4924 treatment for 24 h (mean ± SD, Mann–Whitney U test, n = 3). ∗*p* < 0.05, ∗∗*p* < 0.01, ∗∗∗*p* < 0.001. LDH, lactate dehydrogenase.

Indeed, by inhibiting lactate production, oxamate caused a dose-dependent inhibition of MLN4924-induced H3K18 lactylation (Fig. 3*A*) and largely restored ITGB4 downregulation by MLN4924 at both mRNA and protein levels (Fig. 3, *A* and *B*). Similarly, the siRNA-mediated knockdown of LDHA and LDHB reduced H3K18 lactylation and again largely restored ITGB4 downregulation by MLN4924 (Fig. 3, *C* and *D*). Collectively, ITGB4 is a downregulated gene by MLN4924-induced H3K18 lactylation.

**Figure 4:**
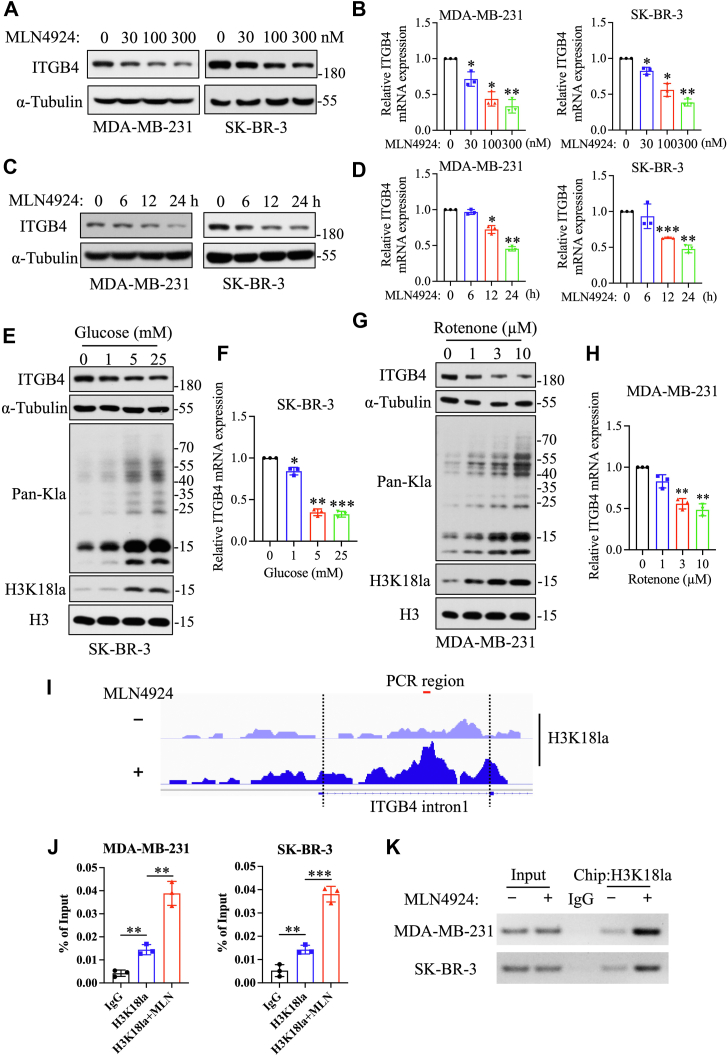
**MLN4924 inhibits ITGB4 expression by enhancing H3K18 lactylation.***A* and *B*, immunoblot (*A*) and qPCR analysis of ITGB4 expression in breast cancer cells treated with indicated concentrations of MLN4924 for 24 h (mean ± SD, Mann–Whitney U test, n = 3) (*B*). *C*, immunoblot analysis of ITGB4 expression in breast cancer cells treated with 100 nM MLN4924 for the indicated time periods. The α-tubulin loading controls shown are the same as those used in Figs. 2*A* and S2*A*, since the data were generated from the same experiment. *D*, qPCR analysis of ITGB4 expression in breast cancer cells treated with 100 nM MLN4924 for indicated time periods (mean ± SD, Mann–Whitney U tests, n = 3). *E*, immunoblot analysis of lactylation levels and ITGB4 protein levels in SK-BR-3 cells cultured with indicated concentrations of glucose for 24 h. *F*, qPCR analysis of ITGB4 mRNA expression in SK-BR-3 cells cultured with indicated concentrations of glucose for 24 h (mean ± SD, Mann–Whitney U test, n = 3). *G*, immunoblot analysis of lactylation levels and ITGB4 protein levels in MDA-MB-231 cells treated with indicated concentrations of rotenone. *H*, qPCR analysis of ITGB4 mRNA expression in MDA-MB-231 cells treated with indicated concentrations of rotenone (mean ± SD, Mann–Whitney U test, n = 3). *I*, IGV track for ITGB4 from CUT&Tag sequencing analysis. *J* and *K*, CHIP-qPCR (*J*) and CHIP-PCR analysis (*K*) of H3K18la enrichment at ITGB4 intron 1 region in breast cancer cells treated with or without 100 nM MLN4924 for 24 h (mean ± SD, unpaired *t* test, n = 3). CUT&Tag, cleavage under target & tagmentation; ∗*p*<0.05, ∗∗*p*<0.01, ∗∗∗*p*<0.001.

The follow-up experiments confirmed that MLN4924 suppressed ITGB4 expression in a dose- and time-dependent manner (Fig. 4, *A*–*D*). Serving as the positive controls, glucose or rotenone, an inhibitor of mitochondrial electron transport (25), increased the lactylation levels of H3K18 as well as nonhistone proteins and downregulated ITGB4 expression in a dose-dependent manner (Figs. 4, *E*–*H* and S4, *A* and *B*). Serving as a negative control, the siRNA-based UBA3 or Cullin-1 knockdown, which failed to affect histone lactylation, also had no effect on ITGB4 expression (Fig. S2, *B* and *C*). Thus, H3K18 lactylation is negatively correlated with ITGB4 expression.

Finally, we investigated how mechanistically H3K18 lactylation downregulates ITGB4 expression. We first cloned a 2-kb promoter region of ITGB4 into the pGL3-basic plasmid, and found that MLN4924 treatment failed to suppress the promoter activity (Fig. S4*C*), indicating that the tested promoter was not subjected to regulation by H3K18 lactylation and that MLN4924 downregulates ITGB4 expression was not through other transcription factors.

We then analyzed CUT&Tag sequencing data and found significant enrichment of the H3K18la signal within intron 1 of ITGB4 following MLN4924 treatment (Fig. 4*I*). This enrichment was confirmed by ChIP-qPCR and ChIP-PCR (Fig. 4, *J* and *K*), suggesting that MLN4924 suppresses ITGB4 transcription by enhancing histone H3K18 lactylation within the intron 1 of the ITGB4 gene.

### MLN4924 suppresses breast cancer cell migration and invasion by downregulating ITGB4 expression

**Figure 5:**
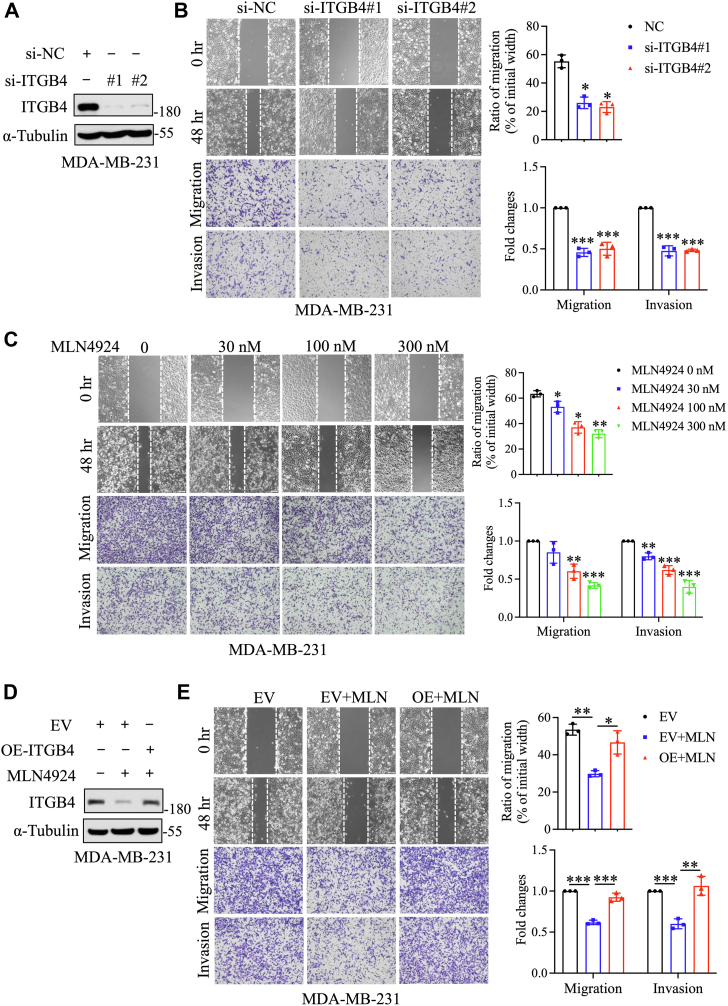
**MLN4924 inhibits migration and invasion of breast cancer cells by downregulating ITGB4 expression.***A*, immunoblot analysis to assess the knockdown efficiency of ITGB4 in MDA-MB-231 cells transfected with siRNA targeting ITGB4 for 48 h *B*, wound-healing assay (*top*) and transwell assay (*bottom*) to investigate the migration and invasion of MDA-MB-231 cells after knocking down ITGB4. Statistical analyses are shown on the *right*. *C*, wound-healing assay (*top*) and transwell assay (*bottom*) to investigate the migration and invasion of MDA-MB-231 cells after treatment with indicated concentrations of MLN4924. Statistical analyses are shown on the *right*. *D*, immunoblot analysis of ITGB4 levels in MDA-MB-231 cells after MLN4924 treatment and transfection with ITGB4 encoding plasmid. *E*, wound-healing assay (*top*) and transwell assay (*bottom*) to investigate the migration and invasion of MDA-MB-231 cells after MLN4924 treatment and ITGB4 overexpression. Statistical analyses are shown on the right. Data are presented as the mean ± SD; statistical significance was assessed by an unpaired *t* test for wound-healing assay and Mann–Whitney U test for transwell assay; n = 3; ∗*p*<0.05, ∗∗*p*<0.01, ∗∗∗*p*<0.001.

ITGB4 has been identified as a key player in promoting the metastasis of various tumor cells (24). To investigate its role in breast cancer cells, we first knocked down ITGB4 *via* siRNA silencing (Figs. 5*A* and S5*A*) and found that capabilities of migration and invasion were significantly inhibited in breast cancer cells (Figs. 5*B* and S5*B*). Moreover, MLN4924, while dose-dependently reducing ITGB4 expression (Fig. 4*A*), also suppressed the migration and invasion of breast cancer cells in a dose-dependent manner (Figs. 5*C* and S5*C*). To further confirm the causal role of ITGB4 in this process, we ectopically expressed ITGB4 in MLN4924-treated breast cancer cells to reach the levels similar to the basal levels prior to MLN4924 treatment (Figs. 5*D* and S5*D*) and found that migration and invasion inhibited by MLN4924 were fully rescued (Figs. 5*E* and S5*E*) in both MDA-MB-231 and SK-BR3 breast cancer cells.

**Figure 6:**
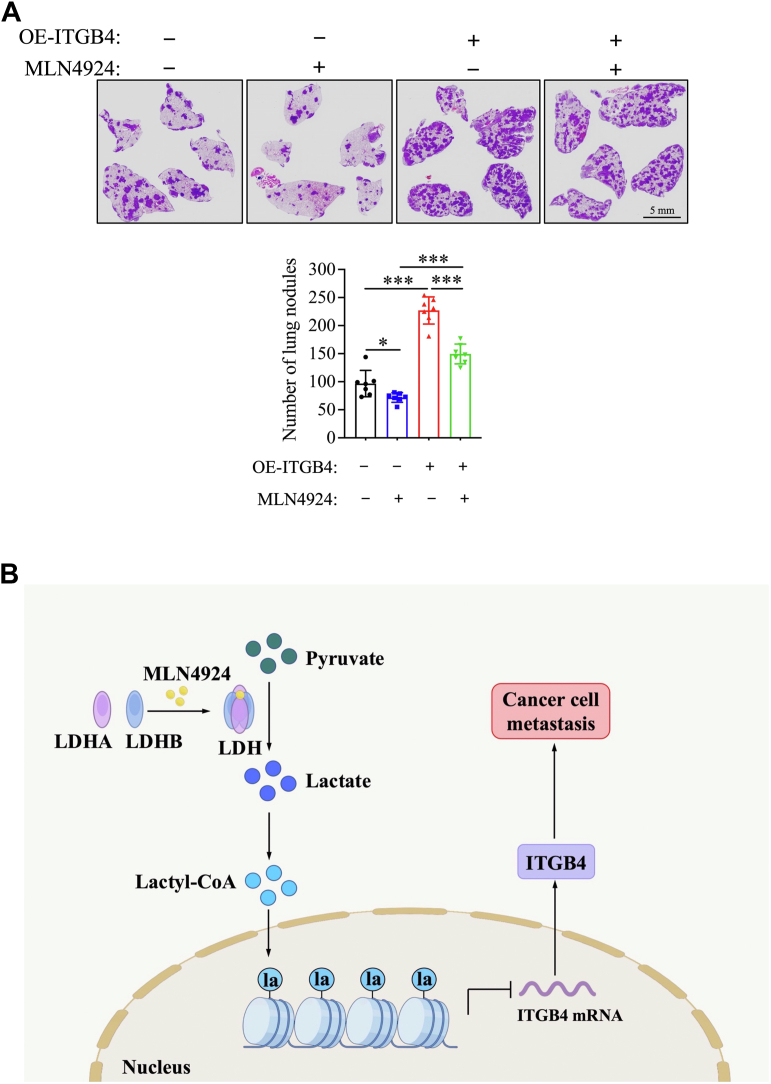
**MLN4924 inhibits *in vivo* metastasis of breast cancer cells by downregulating ITGB4 expression and proposed working model.***A*, representative H&E staining of lung metastatic foci. The number of metastatic nodules in all five lobes of the lungs from each mouse was counted and is shown at the *bottom panel* (mean ± SD, unpaired *t* test, n = 7, ∗*p* < 0.05, ∗∗∗*p* < 0.001). *B*, schematic working model: MLN4924 promotes LDH tetramer formation to increase LDH activity and lactate levels, which increases lactyl-CoA levels to promote histone lactylation, thereby epigenetically downregulating ITGB4 transcription and ultimately inhibiting metastasis of breast cancer cells. LDH, lactate dehydrogenase.

To extend this *in vitro* cell-based observations to an *in vivo* scenario, we next conducted a tail vein injection metastasis assay in BALB/c nude mice using 4T1 murine breast cancer cells. Notably, similar to human breast cancer cells, 4T1 cells also responded to MLN4924 treatment with increased H3K18 lactylation and reduced ITGB4 expression (Fig. S6). In this *in vivo* metastasis model, MLN4924 treatment markedly suppressed lung metastasis in mice injected with control cells, whereas ITGB4 overexpression significantly promoted it, as well as partially reversed the MLN4924 inhibitory effect (Fig. 6*A*). Collectively, these results indicated that MLN4924 effectively suppresses breast cancer cell metastasis, at least in part, by epigenetically downregulating ITGB4 expression *via* inducing H3K18 lactylation.

## Discussion

In this study, we provided significant insights into the novel antimetastatic mechanism of the neddylation inhibitor MLN4924, resulting from its ability to promote histone lactylation and suppress ITGB4 expression in breast cancer cells. Our findings reveal that MLN4924, beyond inhibiting NEDD8-activating enzymes, regulates lactate metabolism and histone modifications, thereby influencing cancer cell behavior.

Histone lactylation was first characterized in 2019, using cell-free system (18). The subsequent studies mainly concentrated on histone H3 lysine 18 lactylation (H3K18la), showing that this modification can activate the transcription of specific genes. For instance, H3K18la has been linked to the upregulation of genes such as c-Myc (26), YTHDF2 (19), YBX1, YY1 (20), platelet-derived growth factor receptor β (21), RUBCNL (27), and POM121 (28). Beyond H3K18la, lactylation at other histone sites has also been implicated in transcriptional activation. For example, elevated H3K9la promoted the transcription of interleukin-11, resulting in CD8+ T cell dysfunction in head and neck squamous cell carcinoma (29), as well as enhanced LUC7L2 transcription, conferring temozolomide resistance in glioblastoma (30). H4K5la promoted PD-L1 expression and drive immunosuppression in acute myeloid leukemia (31), whereas H4K12la activated the transcription of HIF1A, PKM, and LDHA, thereby promoting glycolysis and forming a positive feedback glycolysis/H4K12la/PKM2 loop in microglia (32).

Notably, among all of these histone lactylation events—whether H3K18la, H3K9la, H4K5la, or H4K12la—histone lactylation was shown to enhance the expression of their downstream target genes. Interestingly, however, in this study, we found that MLN4924-induced H3K18la predominantly suppressed gene expression (Fig. 2*B*). An integrative analysis of CUT&Tag sequencing and RNA sequencing data revealed that among the genes with increased H3K18la levels after MLN4924 treatment, only 12 were significantly upregulated, whereas 71 were significantly downregulated. This finding challenges the traditional view of histone lactylation as a purely activating mark. For ITGB4, the gene examined in this study, we observed that MLN4924 enhanced H3K18la specifically in the first intron region, rather than the promoter region, which was the site of histone lactylation for all the previously described targets. This difference in the genomic location of histone lactylation might, at least in part, explain the repressive effect on ITGB4 transcription. To the best of our knowledge, this is the first report demonstrating that histone lactylation can directly suppress gene expression. However, whether this intron-specific lactylation phenomenon extends to other downregulated genes remains to be determined. Further studies are needed to elucidate detailed underlying mechanism for this divergent regulatory role of histone lactylation.

Beyond histone lactylation, lactylation modifications, like other epigenetic marks such as acetylation, also occur on nonhistone proteins. Through integrative lactylome and proteome analysis of hepatitis B virus-related hepatocellular carcinoma cohort, a total of 9256 sites on nonhistone proteins were identified (33). Interestingly, Kla preferentially affected enzymes of metabolic pathways, including the tricarboxylic acid cycle and carbohydrate, amino acid, fatty acid, and nucleotide metabolism. Specifically, lactylation at K28 of adenylate kinase 2 inhibited its activity, thereby facilitating the proliferation and metastasis of hepatocellular carcinoma cells (33). Moreover, p53 was also identified as a target of lactylation. p53 lactylation on lysine 120 and lysine 139 in the DNA-binding domain impairs its liquid–liquid phase separation, DNA binding, and transcriptional activation (34). Other examples included NBS1 lactylation at K388 promoted the formation of the MRE11-RAD50-NBS1 (MRN) complex, whereas MRE11 lactylation at K673 enhanced its DNA binding (35, 36). Both NBS1 and MRE11 lactylation promote homologous recombination–mediated DNA repair. The cGAS lactylation at K131 reversed its DNA-binding function, resulting in the inhibition of cGAMP synthesis and modulation of innate immune signaling (37). In our study, we demonstrated that MLN4924 promotes lactate production by enhancing LDH tetramer formation to activate LDH activity, leading to enhanced lactylation on both histone and nonhistone protein (Fig. 2*A*). Based on these findings, it is plausible that MLN4924 may also promote the lactylation of aforementioned nonhistone proteins, as reported previously. This expanded lactylation landscape could reveal additional roles for MLN4924, further highlighting its functional impact on diverse biological processes and pathways.

Integrins are heterodimeric receptors composed of paired α and β subunits. In the human genome, 18 α and 8 β subunits combine in specific pairings to form 24 integrin receptors, each exhibiting unique specificity for extracellular matrix or cellular adhesion proteins (38). Among these, ITGB4, also known as *CD104*, encodes the integrin β4 subunit, which exclusively pairs with the α6 subunit to form the integrin α6β4 heterodimer. Integrin α6β4 plays a pivotal role in the formation and stabilization of hemidesmosomes, a specialized junctional adhesion complex essential for maintaining epithelial monolayer integrity (39). Furthermore, numerous studies have demonstrated that integrin α6β4 facilitates tumor invasion and metastasis through signaling pathways such as PI3K-Akt (40), FAK-SOX2-HIF1α (41), and Erk-MMP2/MMP9 (42). A recent research reported a novel role for α6β4 in breast cancer metastasis, showing its involvement in abluminal migration along blood vessels to invade the leptomeninges, bypassing the blood–brain barrier (43). The diverse functions of integrin β4, coupled with its pronounced differential expression in various tumors, underscore its potential as a diagnostic marker and therapeutic target (24). In this study, we identify a novel regulatory mechanism in which ITGB4 expression is modulated by histone H3K18 lactylation. Additionally, we demonstrated that MLN4924 significantly suppresses ITGB4 expression and migration and invasion of breast cancer cells, providing new insights into its regulation and potential therapeutic targeting.

In summary, our study demonstrates a novel role of MLN4924 in a manner independent of its canonical activity as an inhibitor of neddylation-activating enzymes. MLN4924 interacts with residues in the binding pocket of LDH to enhance LDH tetramerization and activity, resulting in increased lactate production and histone lactylation. Through integrating CUT&Tag and RNA-seq analyses, we identified ITGB4 as a downstream target of MLN4924-induced histone lactylation. Histone H3K18 lactylation within the first intron region of the ITGB4 gene suppresses its expression and ultimately inhibits *in vitro* migration and invasion and *in vivo* metastasis of breast cancer cells (Fig. 6*B*). These findings highlight a previously unrecognized epigenetic mechanism and provide a compelling basis for further investigation of MLN4924 as a therapeutic agent for cancer metastasis.

## Experimental procedures

### Cell culture and transfection

Human breast cancer cell lines MDA-MB-231, SK-BR-3, and murine breast cancer cell line 4T1 were obtained from the American Type Culture Collection and cultured in the Dulbecco's modified Eagle's medium supplemented with 10% fetal bovine serum and 1% penicillin/streptomycin in a humidified atmosphere at 37 °C with 5% CO2.

Cells were transfected with plasmids or siRNA oligos using Lipofectamine 3000 or Lipofectamine 2000, according to the manufacturer’s instructions, followed by various assays 48 h after transfection. The siRNA sequences were shown in Table S2.

### Immunoblotting and antibodies

Cells were harvested and lysed in the RIPA lysis buffer supplemented with protease inhibitors (Roche, 11873580001) and phosphatase inhibitors (Roche, 04906837001). The protein concentrations were determined using the BCA protein assay kit (Thermo Fisher, 23225). Then equal amounts of total protein lysates were separated by SDS-PAGE and transferred onto PVDF membranes. After blocking with 5% (w/v) milk, the membranes were then incubated with the corresponding primary antibodies. Primary antibodies were used as follows: Pan-Kla (PTM bio, PTM-1401RM), H3K18la (PTM bio, PTM-1427RM), ITGB4 (Abcam, ab182120), LDHA (CST, 3582T), LDHB (Proteintech, 14824-1-AP), Histone H3 (Proteintech, 68,345-1-lg), UBA3 (Abcam, ab124728), CUL-1 (Santa Cruz, sc-11384), and α-Tubulin (Sigma, T8203).

### Quantitative real-time PCR

The total RNA was isolated by TRIzol reagent (Invitrogen, 15596018) and reverse-transcribed to cDNA using PrimeScript 1st Strand cDNA Synthesis kit (Takara, RR037A). The cDNA levels were examined by Applied Biosystems 7900HT Real-Time PCR System using SYBR Premix EX Taq (Takara, RR420A) and normalized to ACTB. The primer sequences are provided in Table S3.

### Measurement of lactate level and LDH activity

Breast cancer cell lines were treated with indicated concentrations of MLN4924 for 24 h, after which cells were harvested. Lactate levels were detected using the Lactic Acid Content Assay Kit (Solarbio, BC2235), and LDH activity was detected using the LDH Activity Assay Kit (Solarbio, BC0685) following the manufacturer’s instructions.

### Detection of LDH tetramer

Breast cancer cell lines were treated with the indicated concentrations of MLN4924 for 24 h. The cells were then harvested and lysed using a nonreducing lysis buffer (50 mM Tris-HCl 7.5, 150 mM NaCl, 1% NP-40, 0.1% SDS, 0.5% sodium deoxycholate, 50 mM NaF, 1 mM EDTA, 1 mM DTT, and 1 mM Na_3_VO_4_). The lysate was then divided into two portions: 0.05% glutaraldehyde was added to the experimental group, while an equal volume of lysis buffer was added to the control group. The samples were incubated at 37 °C for 3 min, and the reaction was neutralized with 10% 1 M Tris-HCl (pH 8.0). Subsequently, 4× SDS loading buffer was added, and the samples were heated at 75 °C for 5 min. After cooling, the samples were subjected to SDS-PAGE followed by immunoblotting using LDHA and LDHB antibodies to assess LDH tetramer formation.

### Molecular docking

Molecular docking is a widely used technique to predict protein–ligand interactions. In this study, molecular docking was performed to predict the binding of MLN4924 to the LDH using AutoDock Vina. The structures of MLN4924 and LDH protein (PDB ID: 6Q0D) were obtained from PubChem and Protein Data Bank databases, respectively. The binding pocket was defined based on the position of original ligand in the protein structure, and the docking pose with the lowest binding energy was selected as the initial pose for subsequent MD simulations.

All of the MD simulations were carried out using AMBER18 package. For each system, ff14SB force field was utilized to model the protein. Partial charges of MLN4924 were determined by the restrained electrostatic potential method at the HF/6-31G∗ level with the Gaussian 09 package, and the Antechamber program with the General Amber force field was used to generate the force field parameters of ligands. Then, Cl^-^ was used to neutralize the system, and 8.0 Å TIP3P water was added, resulting in per periodic cell containing 51,157∼73,512 atoms. Before the product simulation, two stages of minimization were applied: 20,000 steps using the steepest descent algorithm followed by 10,000 steps with the conjugate gradient algorithm. In the first stage of minimization, 500 kcal mol^–1^ Å^–2^ positional restraints were applied for the protein and ligand. Second, the restraints for all atoms were removed. Then, each system was gradually heated to 310 K and reached equilibration after 2 ns simulation. Finally, 100 ns classical MD simulations were performed with the isothermal-isobaric ensemble for each system. The time step was set to 2 fs, and the coordinates were saved every 10 ps.

### SPR analysis

SPR-based measurements were performed by Biacore (8 K). Briefly, the LDHA protein (CUSABIO, CSB-EP012832HU) was immobilized on a CM5 sensorchip (Cytiva) to a level of 7400 response units using Biacore (8 K). For affinity analysis, the short peptides were dissolved in HBS-EP + running buffer (0.1 M Hepes, 1.5 M NaCl, 0.03 M EDTA, 0.5% v/v surfactant P20, and 1% DMSO) at concentrations of 0.39, 0.78, 1.56, 3.125, 6.25, 12.5, and 25uM and were run across the chip. Each sample that was bound to the surface was associated for 90 s at a flow rate of 30 μl/min. Dissociation of sensor chips was performed for 90 s using HBS-EP +. The dissociation constant (K_D_) was determined and recorded using state-steady analysis by Biacore Insight evaluation software (Cytiva).

### Cellular thermal shift assay

Cellular thermal shift assay was performed as previously described (44). SK-BR-3 cells were treated with or without MLN4924 (1 μM) for 24 h. Cells were trypsinized, washed, and resuspended in PBS. Aliquots were heated at 52 to 72 °C in a thermocycler for 3 min. Cells were lysed by three freeze-thaw cycles in liquid nitrogen. The samples were centrifuged, and the supernatants were analyzed by immunoblotting.

### CUT&Tag

CUT&Tag assay was performed to identify genes associated with H3K18 lactylation. MDA-MB-231 cells were treated with or without 100 nM MLN4924 for 24 h. Cells were then harvested, and libraries were constructed using the Hyperactive Universal CUT&Tag Assay Kit (Vazyme, TD903) with an anti-H3K18la antibody, following the manufacturer’s protocol. The resulting libraries were sequenced using Illumina high-throughput sequencing platforms.

### ChIP

For the ChIP assay, breast cancer cells were treated with or without 100 nM MLN4924 for 24 h. Chromatin immunoprecipitation was performed using the SimpleChIP Enzymatic Chromatin IP Kit (CST, 9003) with an anti-H3K18la antibody following the manufacturer’s instructions. Samples were analyzed by real-time PCR using SYBR Premix EX Taq following the manufacturer’s protocol or by RT-PCR with agarose gel electrophoresis. The primers corresponding to ITGB4 intron 1 region are provided in Table S2.

### Dual-luciferase assay

ITGB4 promoter was inserted into pGL3-Basic vector and then cotransfected into breast cancer cells together with pRL-TK. Twelve hours after transfection, cells were treated with 100 nM MLN4924 for 24 h. Luciferase activity was measured with the Dual-Luciferases Reporter Assay kit (Promega E1980), and the ratio of firefly to Renilla luciferase activity was calculated.

### Wound-healing assay

A 10-μl pipette tip was used to create a scratch in the monolayer of cells cultured in 6-well plates. After removing detached cells with PBS, the remaining cells were cultured in serum-free medium. Images of the scratch were taken at 0 h and 48 h after wounding using a Nikon inverted microscope. Gap width was quantified using Image J software, and results represent the mean of three independent experiments.

### Migration and invasion assay

For cell migration, 5 × 10^4^ MDA-MB-231 cells or 1 × 10^5^ SK-BR-3 cells in 100 μl serum-free medium were plated in an 8.0-mm, 24-well plate chamber insert (Corning, 3422), with 10% fetal bovine serum in the lower chamber as a chemoattractant. After 24 h, cells on the upper surface of the insert were removed with a cotton swab, and cells in the bottom chamber were fixed with 4% paraformaldehyde, stained with 0.5% crystal violet, and examined under a microscope. For invasion assays, Matrigel-coated Transwell inserts were used. Cell migration and invasion were quantified using Image J software, and results represent the mean of three independent experiments.

### In vivo metastasis assay

The animal study was approved by and conducted in accordance with the guidelines established by the committee on Use and Care of Animals at the Zhejiang University.

Four-week-old BALB/c athymic nude mice (nu/nu, female) were purchased from Shanghai SLAC Laboratory Animal Center. These mice were housed in the specific pathogen-free environment at a constant temperature (25 °C) and a relatively constant humidity with ad libitum access to water and food (40–60%), with 12 h dark/light cycle.

4T1 cells (2 × 10^5^) stably expressing indicated plasmids were injected *via* tail-vein, the mice were randomized. MLN4924 (30 mg/kg), or vehicle control was given to mice by subcutaneous injection, 5 days a week, for 2 weeks. Mice in the drug control group received 10% 2-hydroxypropyl-β-cyclodextrin as the vehicle control. After 2 weeks, mice were sacrificed, and all the lung tissues were collected, fixed, and sectioned, followed by hematoxylin and eosin staining. Macroscopic metastases were quantified by counting lesions in all five lobes of the lung per mouse.

### Statistical analysis

Statistical analyses were performed using GraphPad Prism 9.0. Data are presented as mean ± SD. Statistical significance was determined using two-tailed Student’s *t* tests or Mann–Whitney U tests, as appropriate. *p* values <0.05 were considered statistically significant (∗*p* < 0.05; ∗∗*p* < 0.01; ∗∗∗*p* < 0.001).

## Data availability

All data that support the findings of this study are available from the corresponding authors upon reasonable request.

## Supporting information

This article contains supporting information.

## Conflict of interest

The authors declare that they have no conflicts of interest with the contents of this article.
