# Supplementary material for: Neddylation inhibitor MLN4924 enhances H3K18 lactylation via binding to LDH and downregulates ITGB4 to block metastasis
Source: J Biol Chem. 2025 Aug 8;301(9):110575. doi: 10.1016/j.jbc.2025.110575 (PMC12446542; doi:10.1016/j.jbc.2025.110575)
Supplement: Supplemental information [file mmc1.pdf]

Supplementary Information for

Neddylation inhibitor MLN4924 enhances H3K18 lactylation via binding to LDH and downregulates ITGB4 to block metastasis

Hongfei Yu, Qiyin Zhou, Yongxia Chen, Changxin Zhong, Tingting Fu, Xiufang Xiong, Feng Zhu, Linbo Wang, and Yi Sun

Correspondence to:

Linbo Wang at linbowang@zju.edu.cn

Yi Sun at yisun@zju.edu.cn

This PDF file includes:

Supplementary figures 1 to 6

Legends for Supplementary figures 1 to 6

Supplementary table 2 and 3

Legend for Supplementary table 1

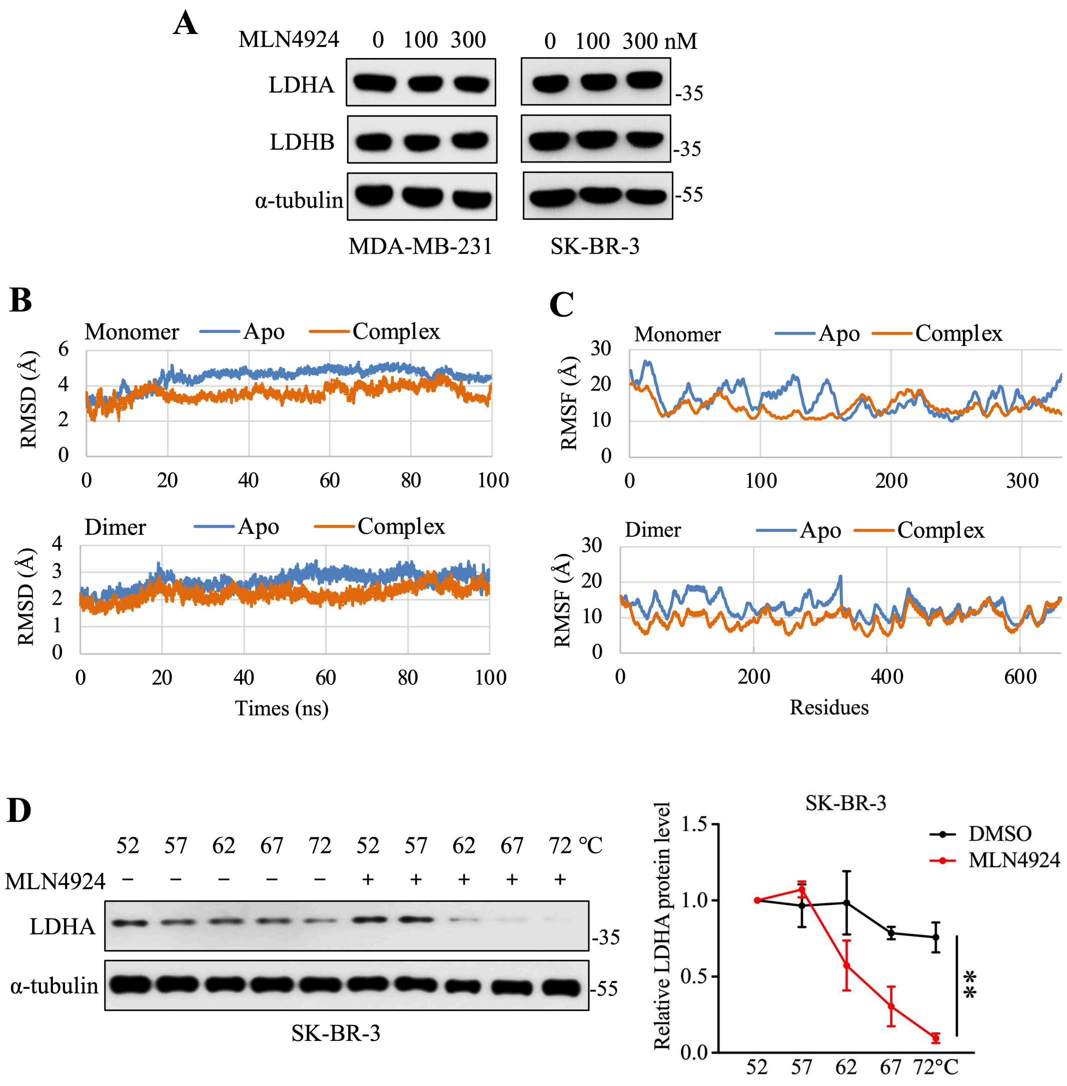

Supplementary Figure 1 MLN4924 promotes LDH tetramer formation. Related to Figure 1. (A) Immunoblot analysis of LDHA and LDHB protein expression levels in breast cancer cells treated with MLN4924 for 24 hours. (B) The RMSD values of protein backbone atoms in the Apo and Complex systems. (C) The RMSF values of protein backbone atoms the Apo and Complex systems. (D) Immunoblotting analysis of thermal stability of LDHA protein by CETSA after treatment of DMSO or 1 μM MLN4924 in SK-BR-3 cells. Statistical analyses are shown on the right (mean ± SEM, unpaired t test, n = 3, ***p*<0.01).

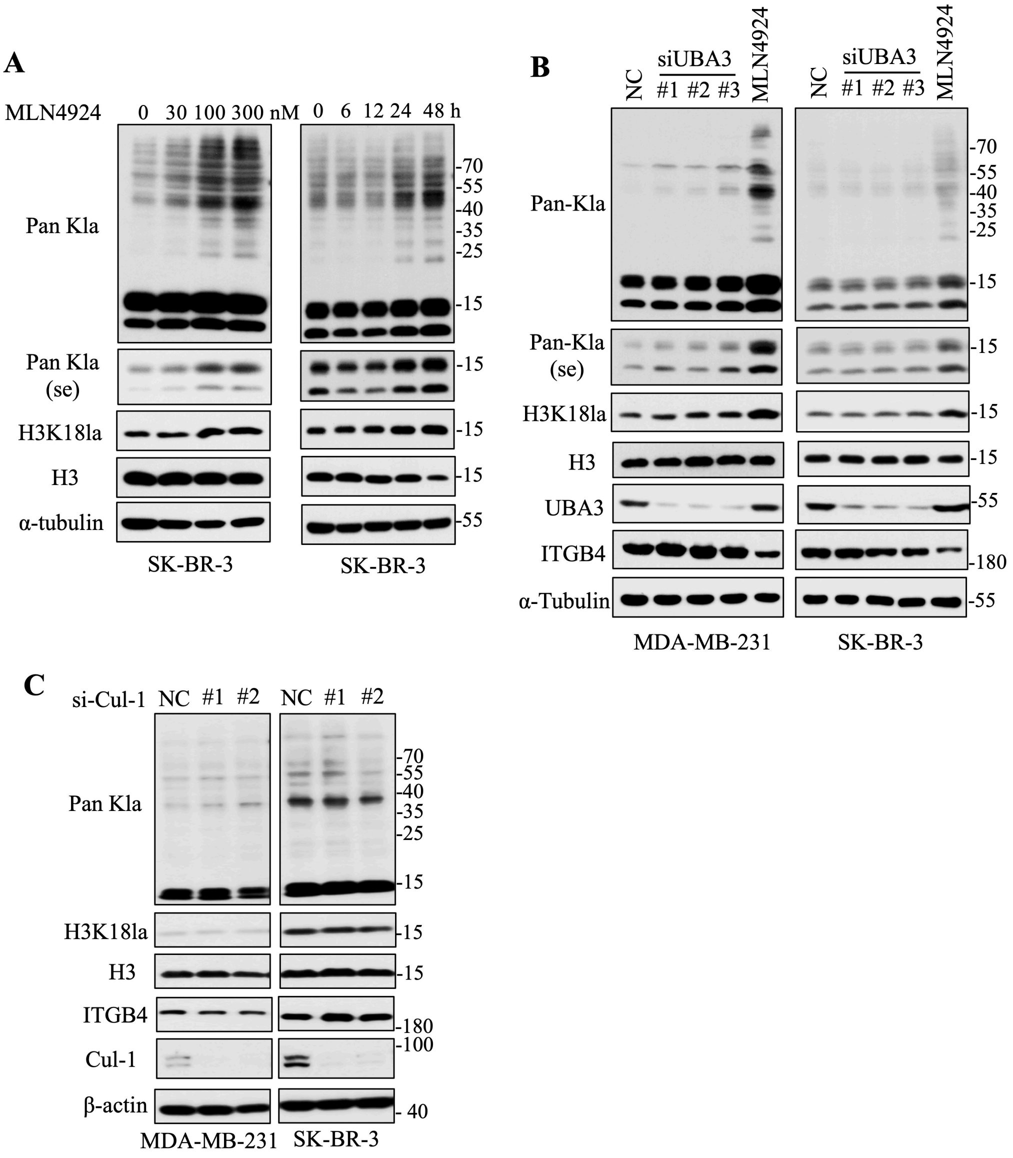

Supplementary Figure 2 MLN4924 promotes histone lactylation. Related to Figure 2.

(A) MLN4924 promotes lactylation in dose- and time-dependent manner. SK-BR-3 cells were treated with indicated concentrations of MLN4924 for 24 hours or indicated time points with 100 nM MLN4924, followed by immunoblotting. Pan-Kla detects lactylation at all lysine sites on proteins. The two lower bands on the top panals represent lactylated histones, while the upper bands represent lactylated non-histone proteins. se: short exposure, only lactylated histones were shown in the short-exposure bands. (B) Immunoblot analysis of UBA3, ITGB4 and Histone lactylation levels following UBA3 knockdown via siRNA for 48 hours or MLN4924 treatment for 24 hours. (C) Immunoblot analysis of Cul-1, ITGB4 and Histone lactylation levels following Cul-1 knockdown via siRNA for 48 hours.

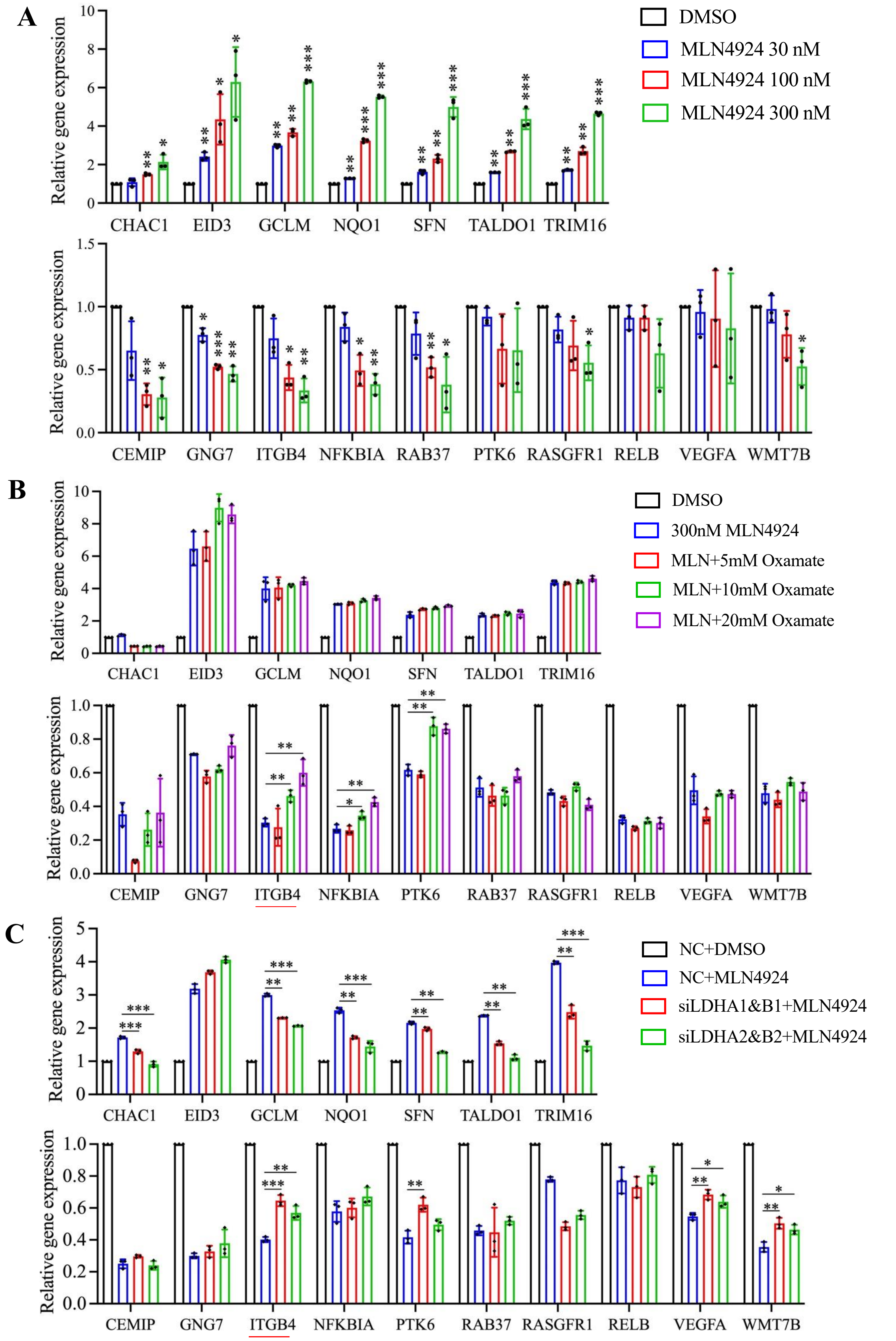

Supplementary Figure 3 Downstream genes regulated by MLN4924-induced histone lactylation. Related to Figure 3.

(A) qPCR analysis of changes in mRNA expression levels of downstream genes in MDA-MB-231 cells after 24 hours of MLN4924 treatment (mean ± SD, Mann–Whitney U test, n = 3). (B) qPCR analysis of changes in mRNA levels of downstream genes in MDA-MB-231 cells treated with MLN4924 and oxamate (mean ± SD, Mann–Whitney U test, n = 3). (C) qPCR analysis of changes in mRNA levels of downstream genes in MDA-MB-231 cells after MLN4924 treatment and knockdown of LDHA and LDHB (mean ± SD, Mann–Whitney U test, n = 3). *p<0.05, **p<0.01, ***p<0.001.

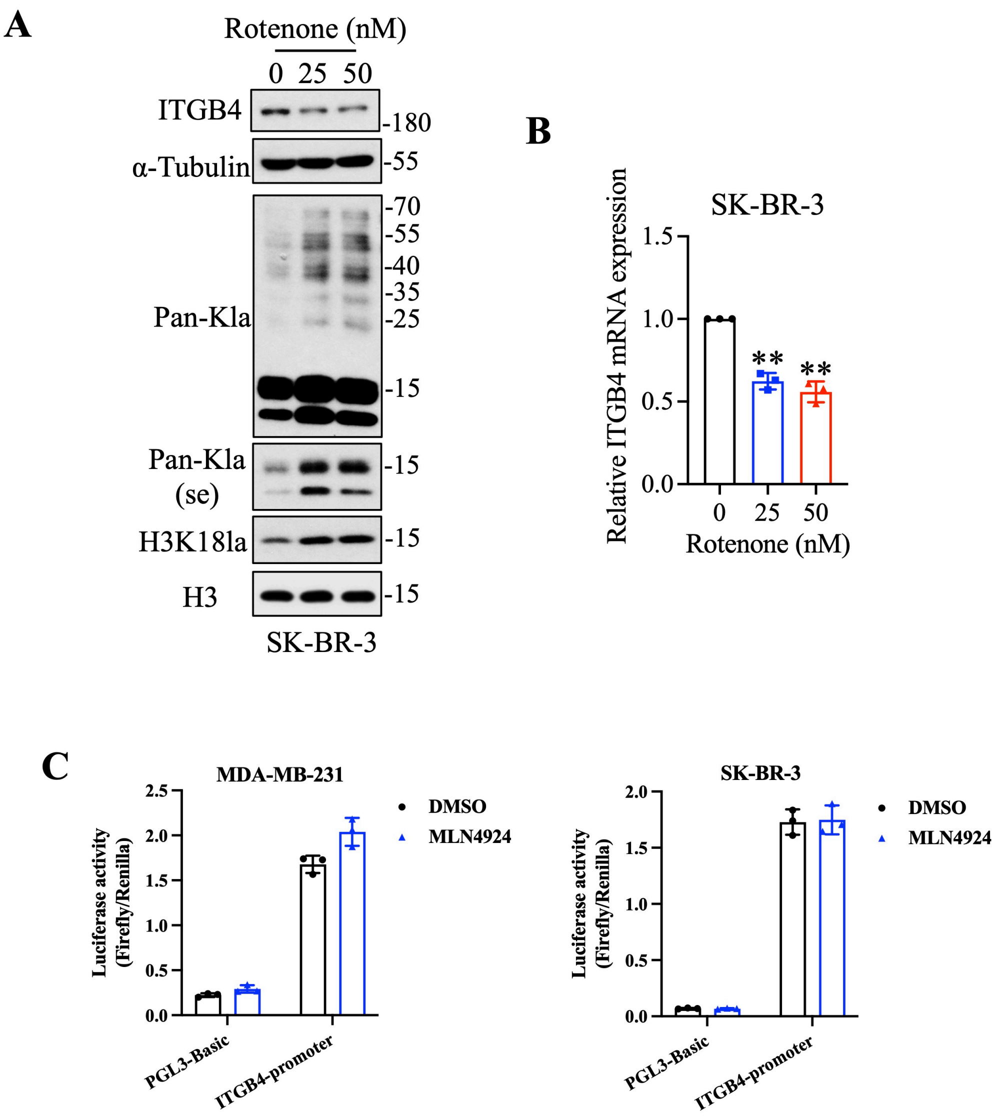

Supplementary Figure 4 MLN4924 inhibits ITGB4 expression by promoting histone lactylation. Related to Figure 4.

(A) Immunoblot analysis of lactylation levels and ITGB4 protein levels in SK-BR-3 cells treated with indicated concentrations of rotenone. (B) qPCR analysis of ITGB4 mRNA expression in SK-BR-3 cells treated with indicated concentrations of rotenone (mean ± SD, Mann–Whitney U test, n = 3, $**p<0.01$). (C) Dual-luciferase reporter assay to evaluate the effect of MLN4924 on ITGB4 promoter activity (mean ± SD, unpaired t test, n = 3).

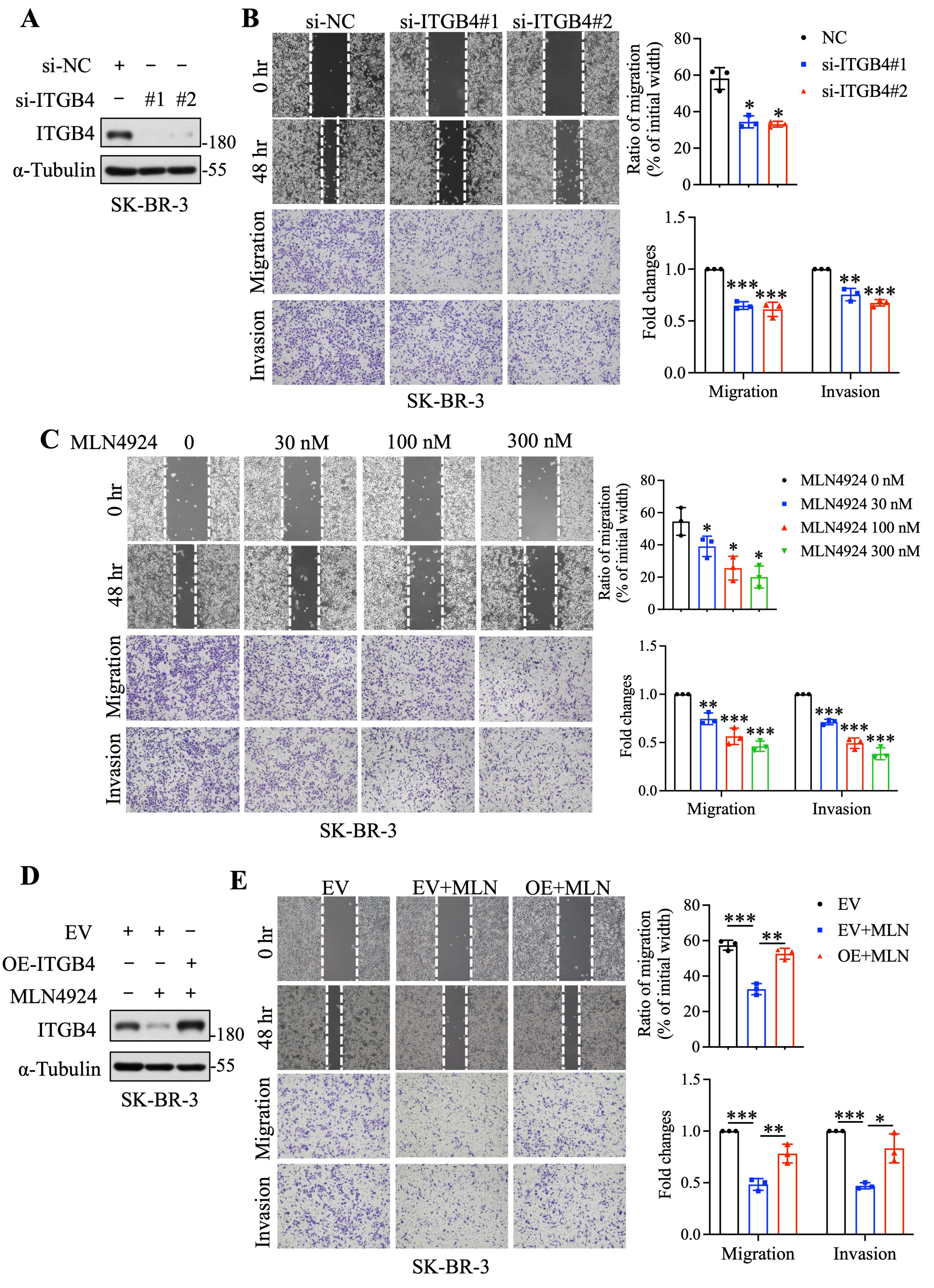

Supplementary Figure 5 MLN4924 inhibits migration and invasion of breast cancer cells by downregulating ITGB4 expression. Related to Figure 5.

(A) Immunoblot analysis to assess the knockdown efficiency of ITGB4 in SK-BR-3 cells transfected siRNA targeting ITGB4 for 48 hours (B) Wound-healing assay (top) and transwell assay (bottom) to investigate the migration and invasion of SK-BR-3 cells after ITGB4 knockdown. Statistical

analyses are shown on the right. (C) Wound-healing assay (top) and transwell assay (bottom) to investigate the migration and invasion of SK-BR-3 cells after treatment with indicated concentrations of MLN4924. Statistical analyses are shown on the right. (D) Immunoblot analysis of ITGB4 levels in SK-BR-3 cells after MLN4924 treatment and transfection with ITGB4 encoding plasmid. (E) Wound-healing assay (top) and transwell assay (bottom) to investigate the migration and invasion of SK-BR-3 cells after MLN4924 treatment and ITGB4 overexpression. Statistical analyses are shown on the right. Data are presented as the mean ± SD; statistical significance was assessed by an unpaired t-test for wound-healing assay and Mann–Whitney U test for transwell assay; n=3. *p<0.05, **p<0.01, ***p<0.001.

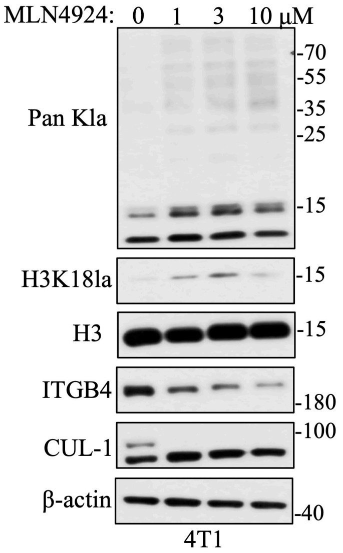

Supplementary Figure 6 Immunoblot analysis of ITGB4 and histone lactylation levels in 4T1 cells following treatment with the indicated concentrations of MLN4924 for 24 hours.

Supplementary Table 1. List of 12 significantly upregulated genes and 71 downregulated genes with enhanced H3K18la after MLN4924 treatment. (The table is shown in another excel file)

Supplementary Table 2 siRNA sequence

Target	Forward	Reverse
ITGB4 Si1	GCGACUACACUAUUGGAUUTT	AAUCCAAUAGUGUAGUCGCTT
ITGB4 Si2	GCUUUAAGGAAGACCACUATT	UAGUGGUCUUCCUUAAAGCTT
LDHA Si1	GGAGAAAGCCGUCUUAAUUTT	AAUUAAGACGGCUUUCUCCTT
LDHA Si2	GGCAAAGACUAUAAUGUAATT	UUACAUUAUAGUCUUUGCCTT
LDHB Si1	AAGAUUGUAGUGGUAACUGCA	UGCAGUUACCACUACAAUCUU
LDHB Si2	GCUUAUUUCUUCAGACACCUA	UAGGUGUCUGAAGAAAUAAGC
UBA3 Si1	GCUUCUCUGCAAAUGAAAUTT	AUUUCAUUUGCAGAGAAGCTT
UBA3 Si2	GCUACCAGAACACUGUAUUTT	AAUACAGUGUUCUGGUAGCTT
UBA3 Si3	CGACACUUUCUAUCGACAATT	UUGUCGAUAGAAAGUGUCGTT
Cul1 Si1	GCUCUACACUCAUGUUUAUTT	AUAAACAUGAGUGUAGAGCTT
Cul1 Si2	GAACCCAGUUACUGAAUAUTT	AUAUUCAGUAACUGGGUUCTT
Negative Control	UUCUCCGAACGUGUCACGUTT	ACGUGACACGUUCGGAGAATT

Supplementary Table 3 qPCR primers sequence

Target	Forward	Reverse
ITGB4	GGCCAATCCCAACAACAACC	AGCCCGGTAGTTGGAATGTG
ACTB	GTCATTCCAAATATGAGATGCGT	GCTATCACCTCCCCTGTGTG
CHAC1	GTGGTGACGCTCCTTGAAGATC	GAAGGTGACCTCCTTGGTATCG
EID3	TCACCGCTGACGAGGAGAAGTG	CTTCCTCCAGAGCCTCGGTTAA
GCLM	TCTTGCCTCCTGCTGTGTGATG	TTGGAAACTTGCTTCAGAAAGCAG
NQO1	CCTGCCATTCTGAAAGGCTGGT	GTGGTGATGGAAAGCACTGCCT
SFN	TGCTGGACAGCCACCTCATCAA	GGCTGAGTCAATGATGCGCTTC
TALDO1	TGCCTGTGCTCTCAGCCAAGG	TTCTCCACAGCCATCTGGTCCT
TRIM16	GGCTGAAGGATAAACTCTCGGG	CGCTGAACAACGGCAGACACTT
CEMIP	CCGAGCGCAGGGAGT	TCAATCCCGGCTTCTATGCG
GNG7	GTGGATGGCGTGAGAGTGAA	GCCAAGGTCTCTCCGTCTTC
NFKBIA	TGCGGGACACGCAGG	TAGTTCACAAGCTCGGGCAG
PTK6	TGTCCATCCCCATCATCCCT	CCCGATGGAGAGCGTAGAAC
RAB37	TTGCCTTGCTGCTCTACCTCCA	GATGGCAGTAGCTGCGCTGATA
RASGRF1	TGTGGTGAGGATCTGCTTCCAG	TCGGCAAATCCGCAGCTCTGAT
RELB	CTGGAGCTCCTGTTTGCCAG	TCGGTGACGATGTCTTGGTG
VEGFA	TCCACTCCATCCTGAAGGCTAC	CAAGGACACCAAAAGCTCCACG
WNT7B	AGAAGACCGTCTTCGGGCAAGA	AGTTGCTCAGGTTCCCTTGGCT
ITGB4-CHIP	CTGAGCCTGCAGCCTGTATT	GGGACAGCAGGGTTCCTAGA
